# Low‐dose exercise protects the heart against established myocardial infarction via IGF‐1‐upregulated CTRP9 in male mice

**DOI:** 10.1002/mco2.411

**Published:** 2023-11-24

**Authors:** Yanzhen Tan, Pan Feng, Lele Feng, Lei Shi, Yujie Song, Jian Yang, Weixun Duan, Erhe Gao, Jincheng Liu, Dinghua Yi, Bing Zhang, Yang Sun, Wei Yi

**Affiliations:** ^1^ Department of Cardiovascular Surgery Xijing Hospital, Fourth Military Medical University Xi'an Shaanxi China; ^2^ Center for Translational Medicine Lewis Katz School of Medicine at Temple University Philadelphia Pennsylvania USA; ^3^ Department of General Medicine Xijing Hospital, Fourth Military Medical University Xi'an Shaanxi China

**Keywords:** CTRP9, established myocardial infarction, exercise, IGF‐1, NR2F2

## Abstract

Regular exercise is recommended as an important component of therapy for cardiovascular diseases in clinical practice. However, there are still major challenges in prescribing an optimized exercise regimen to individual patients with established cardiac disease. Here, we tested the effects of different exercise doses on cardiac function in mice with established myocardial infarction (MI). Exercise was introduced to mice with MI after 4 weeks of surgery. Low‐dose exercise (15 min/day for 8 weeks) improved mortality and cardiac function by increasing 44.39% of ejection fractions while inhibiting fibrosis by decreasing 37.74% of distant region. Unlike higher doses of exercise, low‐dose exercise consecutively upregulated cardiac expression of C1q complement/tumor necrosis factor‐associated protein 9 (CTRP9) during exercise (>1.5‐fold). Cardiac‐specific knockdown of CTRP9 abolished the protective effects of low‐dose exercise against established MI, while cardiac‐specific overexpression of CTRP9 protected the heart against established MI. Mechanistically, low‐dose exercise upregulated the transcription factor nuclear receptor subfamily 2 group F member 2 by increasing circulating insulin‐like growth factor 1 (IGF‐1), therefore, upregulating cardiac CTRP9 expression. These results suggest that low‐dose exercise protects the heart against established MI via IGF‐1‐upregulated CTRP9 and may contribute to the development of optimized exercise prescriptions for patients with MI.

## Graphical abstract

Low‐dose exercise exerted cardioprotective effects against established MI in mice. Mechanistically, low‐dose exercise upregulated the transcription factor nuclear receptor subfamily 2 group F member 2 (NR2F2) by increasing circulating insulin‐like growth factor 1 (IGF‐1), therefore, upregulating cardiac C1q complement/tumor necrosis factor‐associated protein 9 (CTRP9) expression.

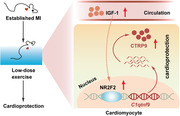

## 1 INTRODUCTION

Regular exercise has been well established to provide extensive beneficial effects on human health, including reduced cardiovascular and all‐cause mortality rates.^1^, ^2^, ^3^, ^4^ It is an attractive treatment option for almost all cardiovascular diseases (CVDs) and is recommended as an important component of therapy for CVDs in clinical practice.^5^ However, patients with CVDs often experience exercise intolerance, and exercise may paradoxically trigger adverse events such as sudden cardiac arrest and acute coronary syndrome, particularly in this population.^6^ Despite significant progress, there are still major challenges in prescribing an optimized regimen of exercise to individual patients.^7^

Myocardial infarction (MI) occurs when coronary blood flow is occluded and is a major contributor to total cardiovascular mortality worldwide.^8^ Emerging evidence has confirmed that exercise after MI prevents future complications and increases the quality of life and longevity of patients when adequately prescribed and supervised.^9^ However, some clinical studies have reported negative effects of exercise in patients with coronary artery diseases or heart failure. This highlights the need for more evidence on the optimal exercise regimen for MI treatment.^10^ The biological effects of exercise largely depend on its dose, but the specific exercise dose that should be prescribed to patients with MI remains unclear. Therefore, there is a need for experimental studies to explore the biological effects of different exercise regimens on MI and the underlying mechanisms to provide evidence for optimized prescription of exercise for patients with MI. Previous studies examining the biological effects of exercise on MI have mainly focused on exercise interventions either before MI (exercise preconditioning) or immediately after MI.^11^ However, as the progression of MI is complex and exercise intervention at different time points may exert different biological effects, the current exercise intervention strategy before or at the early phase of MI lacks clinical relevance. Hence, it is essential to investigate the effects of exercise interventions on established MI or at the late phase of MI.

The C1q complement/tumor necrosis factor‐associated proteins (CTRPs) superfamily is a paralog of adiponectin, composed of CTRP1–CTRP15. Recent studies have highlighted the critical role of CTRPs in the pathophysiology of CVDs.^12^, ^13^ These proteins are involved in regulating endothelial function, inflammatory response, metabolism, and other important processes.^13^ Our previous studies have demonstrated the robust cardioprotective effects of C1q complement/tumor necrosis factor‐associated protein 9 (CTRP9), including its ability to improve cardiac function following MI, reduce myocardial fibrosis, and decrease mortality rates associated with MI.^14^

In this study, we tested the effects of different doses of exercise on cardiac function in mice with established MI and explored whether CTRPs underlie the exercise‐afforded beneficial effects. Exercise of different intensities was introduced to mice with MI after 4 weeks of surgery. CTRP9 levels wer measured, followed by cardiac‐specific knockdown or cardiac‐specific overexpression of CTRP9 to explore its role in exercise‐mediated protection of the heart against established MI. The mechanism of exercise increasing CTRP9 was studied. We aimed to provide valuable insights into the biological effects and underlying mechanisms of exercise in the context of MI, which may contribute to the development of optimized exercise prescriptions for patients with MI.

## 2 RESULTS

### 2.1 Low‐dose exercise protects the heart against established MI

**FIGURE 1:**
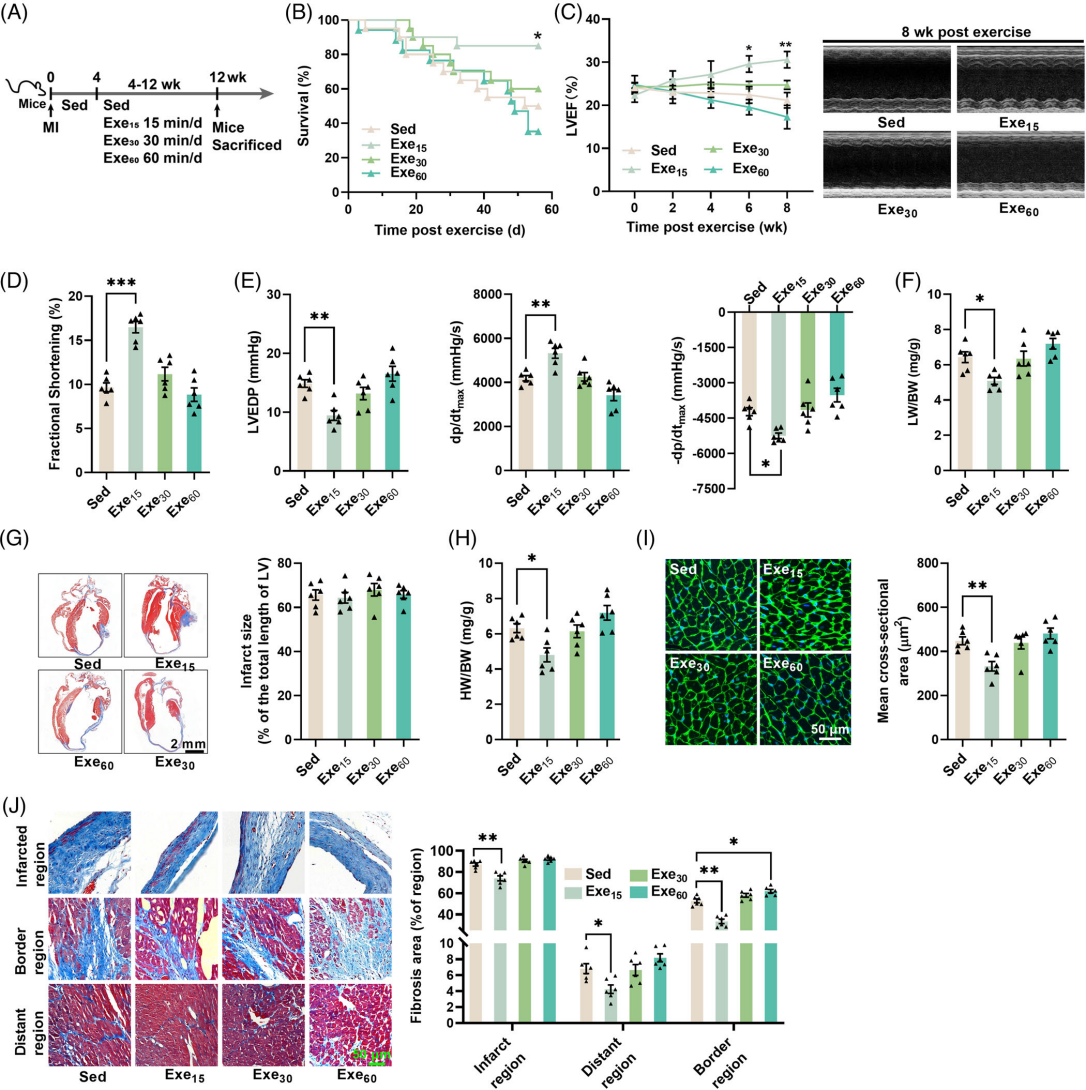
Low‐dose exercise protects the heart against established myocardial infarction (MI). (A) The exercise training protocol for mice with established MI. MI mice (4 weeks post‐surgery) were subjected to 15‐, 30‐, or 60‐min swimming training every day for 8 weeks. (B) Effects of exercise on 8‐week survival of mice with MI (*n* = 20), ^*^ *p* < 0.05 versus Sed group. (C and D) Effects of exercise on cardiac systolic function (*n* = 6), ^*^ *p* < 0.05, ^**^ *p* < 0.01 versus Sed group. (E) Low‐dose exercise improved cardiac function as detected by a micromanometer‐tipped catheter (*n* = 6). (F) Lung weight to body weight ratio (*n* = 6). (G) Infarct area of mice heart with established MI (*n* = 6). (H) Heart weight to body weight ratio (*n* = 6) of mice with established MI. (I and J) Cardiac wheat germ agglutinin (WGA) staining (*n* = 6) and Masson staining (*n* = 6) of mice with established MI. ^*^ *p* < 0.05, ^**^ *p* < 0.01, ^***^ *p* < 0.001.

Mice with established MI (4 weeks post‐surgery) were subjected to different doses of swimming exercises (0, 15, 30, and 60 min/day for 8 weeks) (Figure 1A). Only low‐dose exercise (15 min/day) led to significant improvements in survival and cardiac function (Figure 1B–E). It improved cardiac systolic function, as evidenced by an increase in left ventricle (LV) ejection fraction and fractional shortening, a decrease in LV end‐diastolic pressure (LVEDP), and an increase in ±LV d*P*/d*t*_max_ (Figure 1C–E). Although low‐dose exercise decreased the lung weight to body weight ratio (Figure 1F), all exercise interventions had no significant effects on infarct size in mice with established MI (Figure 1G). Low‐dose exercise also reduced heart weight to body weight ratio, the size of cardiomyocytes and cardiac fibrosis (Figure 1H–J). In contrast, moderate‐dose exercise (30 min/day) and high‐dose exercise (60 min/day) had no significant effects on both cardiac structure and function in mice with established MI (Figure 1B–J). Surprisingly, high‐dose exercise increased cardiac fibrosis in the border region of the heart in mice with established MI (Figure 1J). These results suggest that only low‐dose exercise can protect the heart against established MI.

### 2.2 Low‐dose exercise upregulates myocardial CTRP9 expression in mice with established MI

**FIGURE 2:**
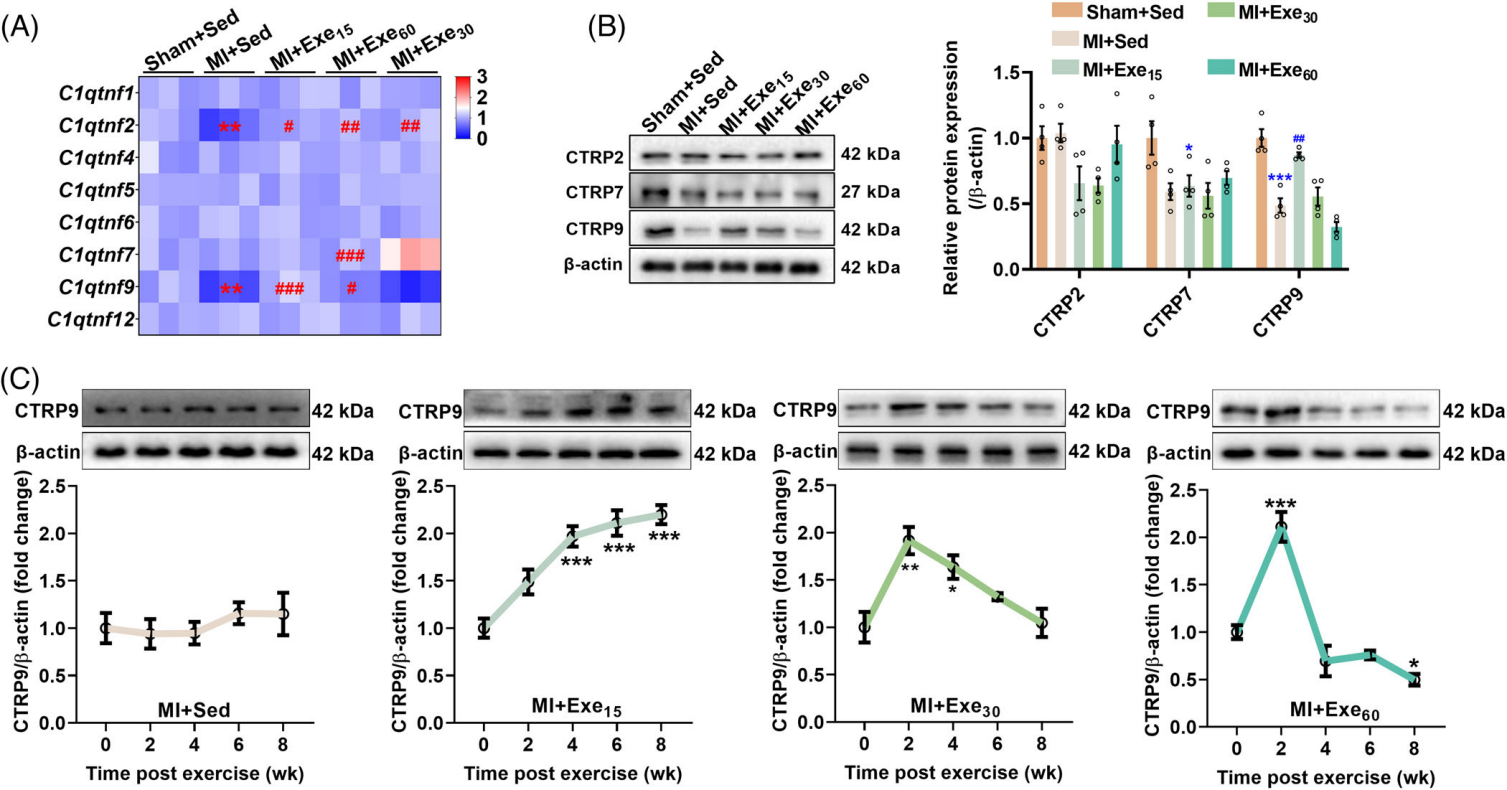
Low‐dose exercise consecutively upregulates myocardial C1q complement/tumor necrosis factor‐associated protein 9 (CTRP9) expression in mice with established myocardial infarction (MI). (A) Cardiac transcriptional levels of CTRPs in MI mice after swimming training for 8 weeks (*n* = 3), ^**^ *p* < 0.01 versus Sham + Sed group, **^#^** *p* < 0.05, **^##^** *p* < 0.01, **^###^** *p* < 0.001 versus MI + Sed group. (B) Cardiac protein levels of CTRP2, CTRP7, and CTRP9 in MI mice after swimming training for 8 weeks (*n* = 4), ^*^ *p* < 0.05, ^***^ *p* < 0.001 versus Sham + Sed group, **^##^** *p* < 0.01 versus MI + Sed group. (C) Cardiac protein levels of CTRP9 in MI mice during swimming training (*n* = 4). ^*^ *p* < 0.05, ^**^ *p* < 0.01, ^***^ *p* < 0.001 versus 0‐week group.

To investigate whether CTRPs are involved in exercise‐induced cardioprotection, we measured the transcriptional levels of CTRPs. MI reduced the transcriptional levels of *C1qtnf2* and *C1qtnf9*, while low‐ and moderate‐dose exercise upregulated their transcriptions. High‐dose exercise upregulated the transcriptional levels of *C1qtnf2* and *C1qtnf7* (Figure 2A). At the protein level, MI reduced CTRP7 and CTRP9 contents, and low‐dose exercise upregulated CTRP9 content, indicating a potential role of CTRP9 in exercise‐induced cardioprotection (Figure 2B). To further investigate the temporal regulation of CTRP9 expression by exercise, we detected its expression during the progression of MI. CTRP9 expression was not changed during the progression of MI (Figure 2C), while low‐dose exercise consecutively upregulated CTRP9 expression, and moderate‐ and high‐dose exercise transiently upregulated CTRP9 expression (Figure 2C). These findings suggested that CTRP9 may play a critical role in low‐dose exercise‐afforded cardioprotection against established MI.

### 2.3 Cardiac‐specific knockdown of CTRP9 abolishes the protective effects of low‐dose exercise on established MI

**FIGURE 3:**
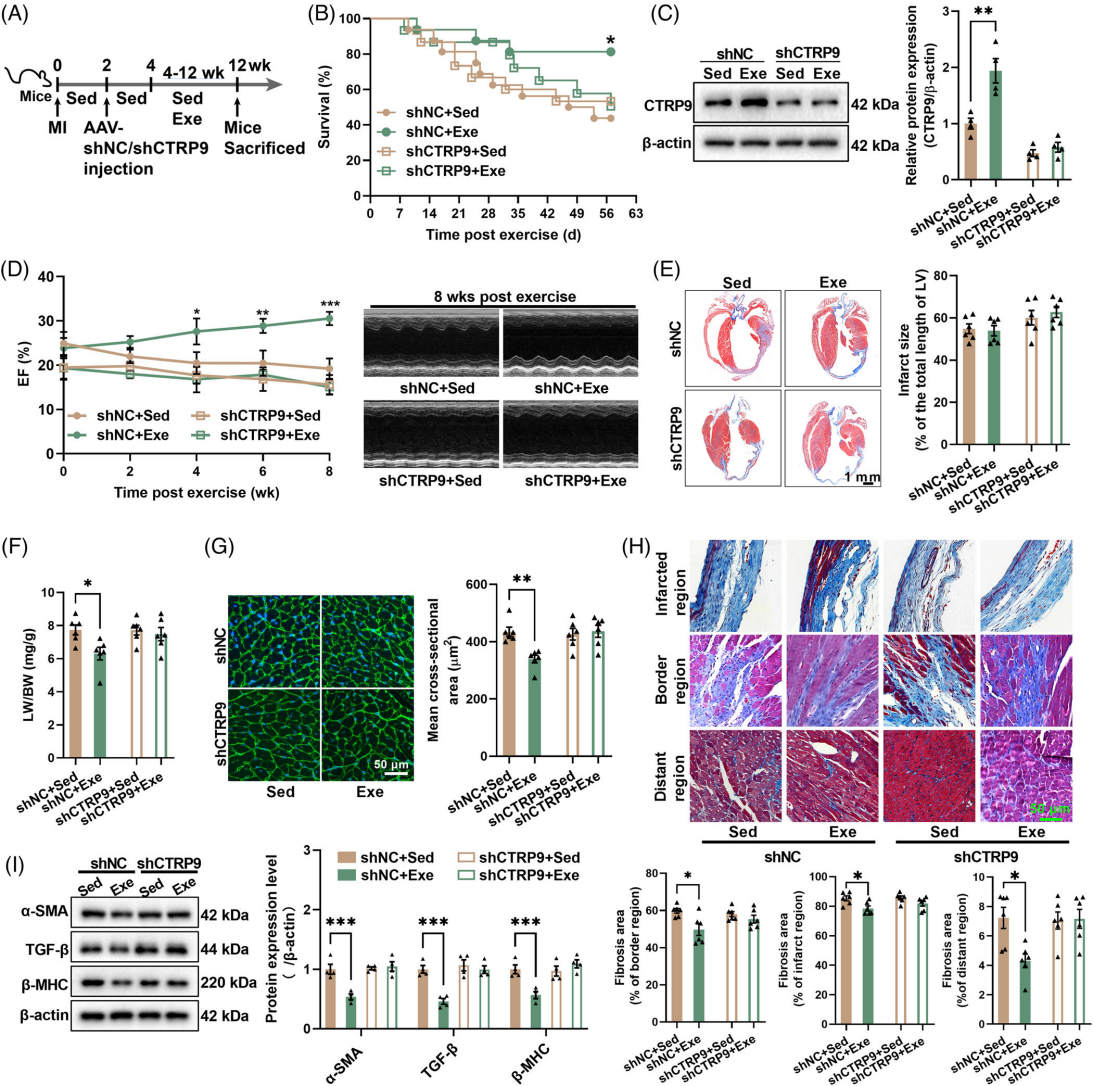
Cardiac‐specific knockdown of C1q complement/tumor necrosis factor‐associated protein 9 (CTRP9) abolishes the protective effects of low‐dose exercise on established myocardial infarction (MI). (A) The protocol for exercise training in mice with cardiac‐specific knockdown of CTRP9 and established MI. AAV9 carrying shRNA negative control (shNC) or shCTRP9 was administered via tail vein injection 2 weeks post‐surgery. MI mice (4 weeks post‐surgery) were subjected to 15 min of swimming training every day for 8 weeks. (B) Effects of exercise on 8‐week survival in cardiac‐specific CTRP9 knockdown mice with established MI (*n* = 16), ^*^ *p* < 0.05 versus shNC + Sed. (C) Cardiac CTRP9 contents in mice post‐exercise (*n* = 4). (D) Effects of exercise on cardiac systolic function in CTRP9 knockdown mice with established MI (*n* = 8), ^*^ *p* < 0.05, ^**^ *p* < 0.01, ^***^ *p* < 0.001 versus shNC + Sed. (E and F) Cardiac infarct area (E) (*n* = 6) and lung weight to body weight ratio (F) (*n* = 6) in CTRP9 knockdown mice with established MI. (G and H) Cardiac wheat germ agglutinin (WGA) staining (G) (*n* = 6) and Masson staining (H) (*n* = 6) in CTRP9 knockdown mice with established MI. (I) Cardiac protein contents of α‐SMA, TGF‐β, and β‐MHC in CTRP9 knockdown mice with established MI (*n* = 4). ^*^ *p* < 0.05, ^**^ *p* < 0.01, ^***^ *p* < 0.001.

To determine whether CTRP9 is involved in the low‐dose exercise‐induced cardioprotection, we introduced cardiac‐specific knockdown of CTRP9 using AAV9. CTRP9 knockdown abolished the effects of low‐dose exercise on survival and cardiac CTRP9 expression in mice with established MI (Figure 3A–C). Consequently, it also abolished the effects of exercise on cardiac structure and function, as evidenced by unchanged LV ejection fraction, infarct size, cardiomyocyte size, and fibrosis induced by low‐dose exercise in CTRP9 knockdown mice (Figure 3D–I). Moreover, we observed little effect on the lung weight to body weight ratio in CTRP9 knockdown mice (Figure 3F). Additionally, in control mice with established MI, low‐dose exercise decreased the expression of α‐smooth muscle actin (α‐SMA), transforming growth factor beta (TGF‐β), and β‐myosin heavy chain (β‐MHC), but had little effect on their contents in CTRP9 knockdown mice with established MI (Figure 3I). These results suggest that low‐dose exercise protects the heart against established MI by upregulating cardiac CTRP9.

### 2.4 Cardiac‐specific overexpression of CTRP9 protects the heart against established MI

**FIGURE 4:**
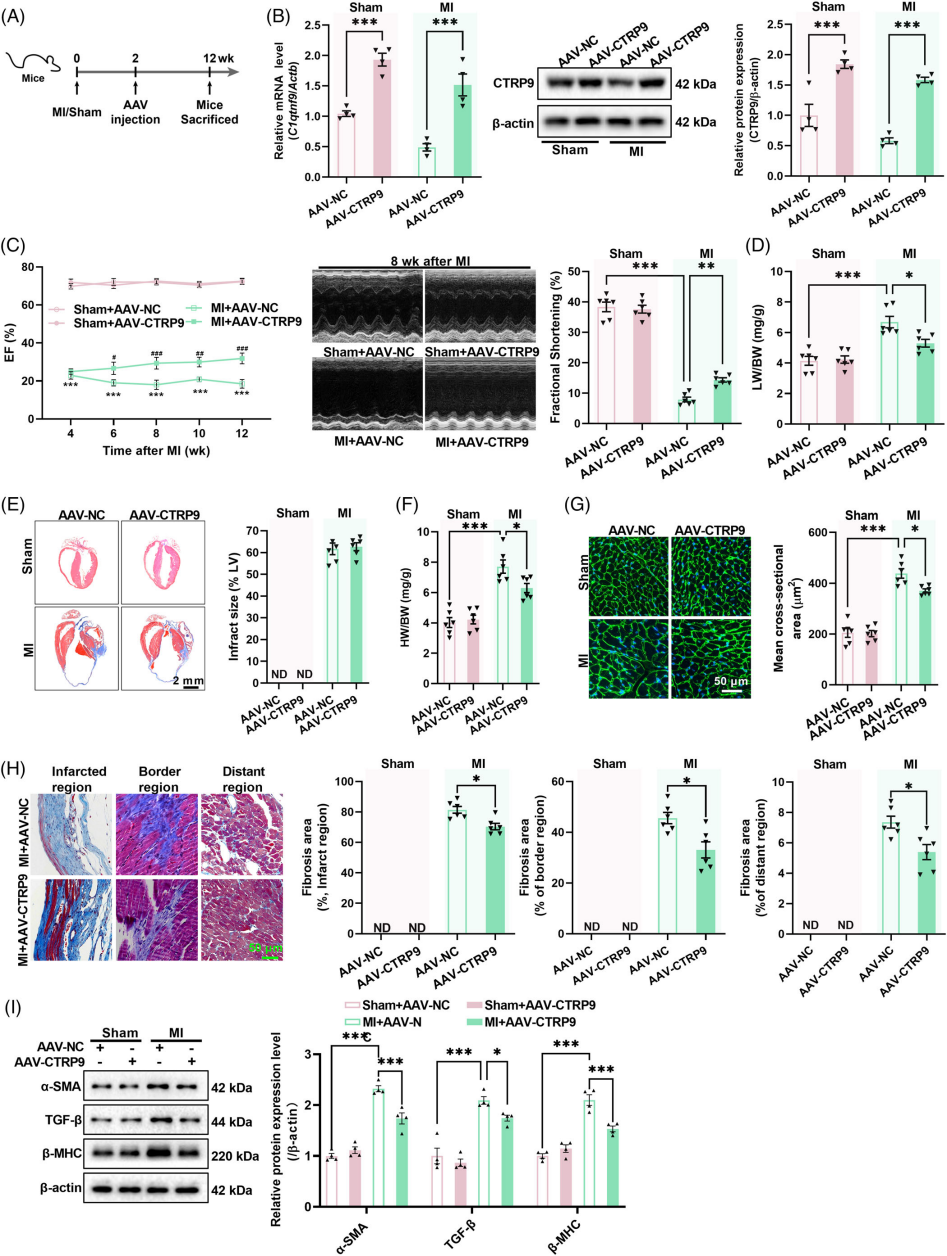
Cardiac‐specific overexpression of C1q complement/tumor necrosis factor‐associated protein 9 (CTRP9) protects the heart against established myocardial infarction (MI). (A) The protocol for cardiac‐specific overexpression of CTRP9 in mice with established MI. AAV9 carrying negative control (NC) or CTRP9 gene was administered via tail vein injection 2 weeks post‐surgery. (B) CTRP9 levels in the mouse heart 12 weeks after MI (*n* = 4). (C–F) Effects of cardiac‐specific overexpression of CTRP9 on cardiac ejection fraction (C) (*n* = 8), ^***^ *p* < 0.001 versus Sham + AAV‐NC group, ^#^ *p* < 0.05, ^##^ *p* < 0.01, ^###^ *p* < 0.001 versus MI + AAV‐NC group, and fractional shortening (C) (*n* = 6), lung weight to body weight ratio (D) (*n* = 6), cardiac infarct area (E) (*n* = 6), and heart weight to body weight ratio (F) (*n* = 6). (G and H) Cardiac wheat germ agglutinin (WGA) staining (G) (*n* = 6) and Masson staining (H) (*n* = 6) in mice with established MI. (I) Cardiac contents of α‐SMA, TGF‐β, and β‐MHC in mice with established MI (*n* = 4). ^*^ *p* < 0.05, ^**^ *p* < 0.01, ^***^ *p* < 0.001.

To determine whether CTRP9 overexpression protects the heart against established MI, CTRP9 was overexpressed in the heart (Figure 4A,B). In sham mice, cardiac‐specific overexpression of CTRP9 showed little effect on cardiac structure and function (Figure 4C–I). However, in mice with established MI, cardiac‐specific overexpression of CTRP9 increased LV ejection fraction and fractional shortening (Figure 4C) while decreasing the lung weight to body weight ratio and heart weight to body weight ratio, although it did not affect infarct size (Figure 4D–F). Furthermore, cardiac‐specific overexpression of CTRP9 decreased cardiomyocyte size, cardiac fibrosis, and the cardiac contents of α‐SMA, TGF‐β, and β‐MHC in mice with established MI (Figure 4G–I). These results suggest that upregulation of cardiac CTRP9 can protect the heart against established MI.

### 2.5 Low‐dose exercise upregulates cardiac CTRP9 expression by increasing circulating insulin‐like growth factor 1

**FIGURE 5:**
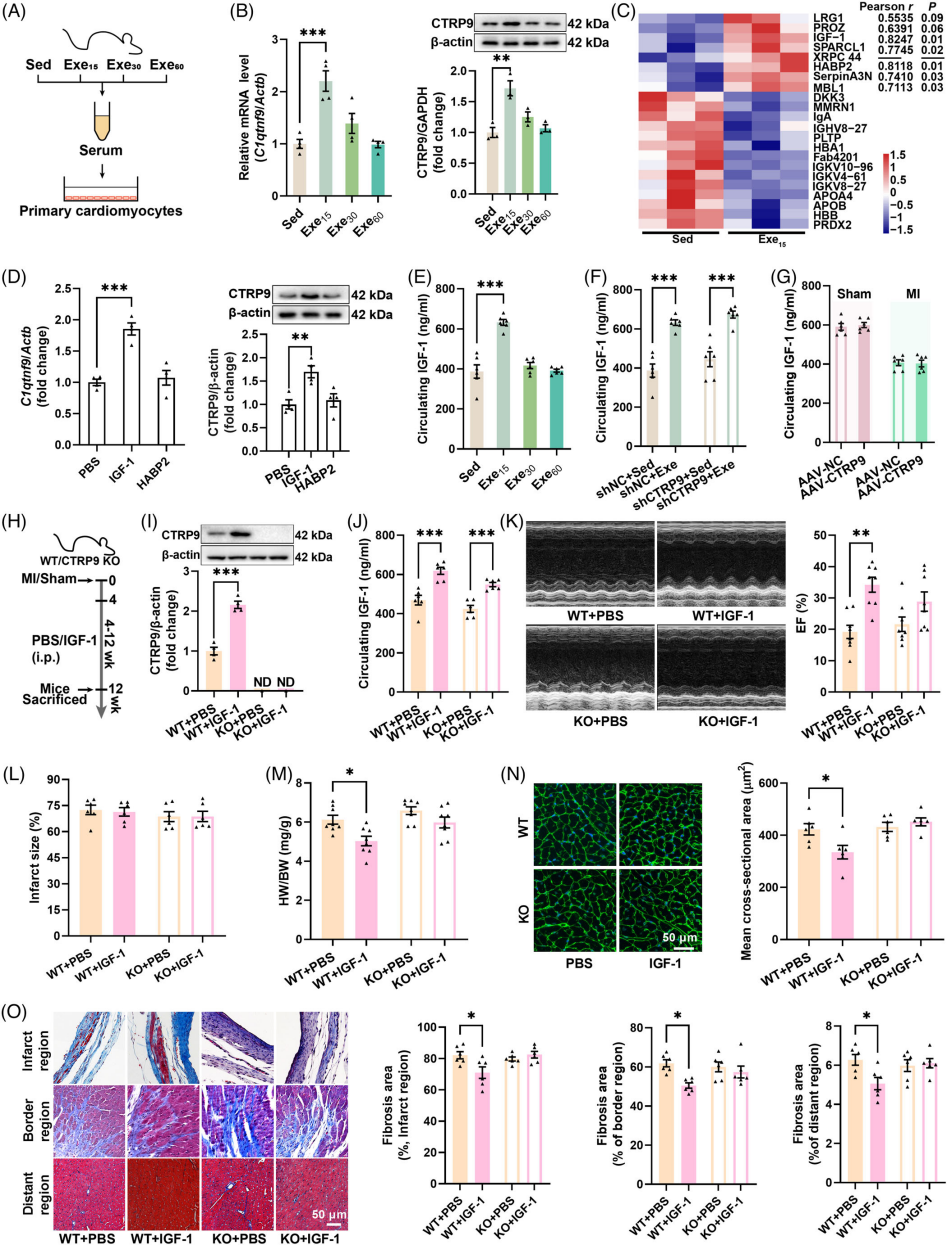
Low‐dose exercise upregulates cardiac C1q complement/tumor necrosis factor‐associated protein 9 (CTRP9) expression by increasing circulating insulin‐like growth factor 1 (IGF‐1). (A) Isolated cardiomyocytes were treated with serum samples from myocardial infarction (MI) mice with different doses of exercise intervention. (B) CTRP9 expression level in cardiomyocytes after treatment with serum from MI mice (*n* = 4) for quantitative real‐time polymerase chain reaction (qPCR) and *n* = 3 for western blotting. (C) Heatmap of serum proteomics of MI mice with low‐dose exercise (*n* = 3). (D) CTRP9 expression level in cardiomyocytes after treatment with IGF‐1 or hyaluronan binding protein 2 (HABP2) (*n* = 4). (E) Concentration of circulating IGF‐1 in MI mice post‐exercise (*n* = 6). (F) Concentration of circulating IGF‐1 in cardiac‐specific knockdown of CTRP9 mice post‐exercise (*n* = 6). (G) Concentration of circulating IGF‐1 in cardiac‐specific CTRP9 overexpression mice (*n* = 6). (H) Protocol for IGF‐1 supplementation in CTRP9 knockout (KO) mice with established MI. IGF‐1 was administered via intraperitoneal injection 4 weeks post‐surgery. (I) CTRP9 expression level in CTRP9 KO mice heart post‐exercise (*n* = 4). (J) Concentration of circulating IGF‐1 in CTRP9 KO mice after administration of IGF‐1 (*n* = 6). (K) Supplementation of IGF‐1 failed to improve cardiac function in CTRP9 KO mice (*n* = 8). (L and M) Cardiac infarct size (L) (*n* = 6) and heart weight to body weight ratio (M) (*n* = 8) in CTRP9 KO mice with IGF‐1 treatment. (N and O) Cardiac wheat germ agglutinin (WGA) staining (N) (*n* = 6) and Masson staining (O) (*n* = 6) in CTRP9 KO mice with IGF‐1 treatment. ^*^ *p* < 0.05, ^**^ *p* < 0.01, ^***^ *p* < 0.001.

To investigate whether exercise‐induced upregulation of cardiac CTRP9 is mediated by circulating factors, isolated cardiomyocytes were treated with serum from sedentary and exercised mice. Serum from low‐dose exercise mice was found to upregulate both the transcriptional and protein levels of CTRP9 in isolated cardiomyocytes (Figure 5A,B), indicating a potential role of circulating factors in the regulation of cardiac CTRP9 expression. Proteomics analysis of serum samples from sedentary and low‐dose exercise mice revealed seven upregulated circulating proteins and the contents of insulin‐like growth factor 1 (IGF‐1) and hyaluronan binding protein 2 (HABP2) were highly associated with the expression of cardiac CTRP9 in mice with established MI (Figure 5C). Treatment of cardiomyocytes with IGF‐1 or HABP2 showed that IGF‐1 upregulated both CTRP9 transcription and expression (Figure 5D). Moreover, low‐dose exercise upregulated circulating IGF‐1 content in mice with established MI (Figure 5E). Low‐dose exercise also upregulated circulating IGF‐1 contents in both control and CTRP9 knockdown mice, whereas CTRP9 overexpression showed no significant effects on circulating IGF‐1 contents in mice with established MI (Figure 5F,G). To test whether IGF‐1 contributes to CTRP9 upregulation in the heart, mice with established MI were treated with IGF‐1 for 8 weeks (Figure 5H). IGF‐1 treatment increased circulating IGF‐1 contents in both wild‐type (WT) and CTRP9 knockout (KO) mice but only upregulated cardiac CTRP9 expression in WT mice (Figure 5I,J). Additionally, IGF‐1 treatment improved cardiac function in WT but not CTRP9 KO mice without affecting infarct size (Figure 5K,L). It also decreased heart weight to body weight ratio, cardiomyocyte size, and cardiac fibrosis in WT mice but not in CTRP9 KO mice with established MI (Figure 5M–O). These results suggest that low‐dose exercise upregulates cardiac CTRP9 expression through the upregulation of circulating IGF‐1.

### 2.6 Circulating IGF‐1 upregulates cardiac CTRP9 through upregulation of NR2F2

**FIGURE 6:**
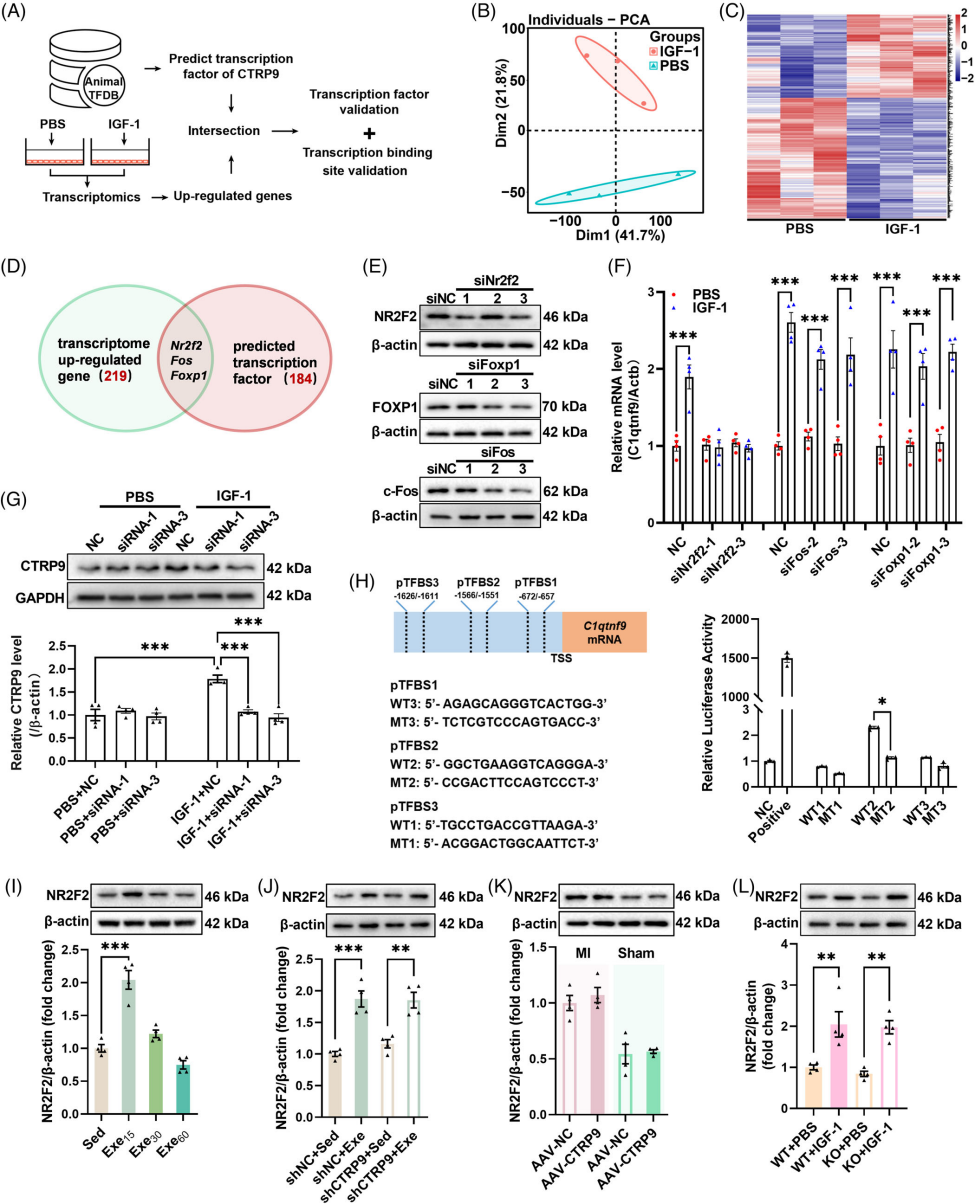
Circulating insulin‐like growth factor 1 (IGF‐1) upregulates cardiac C1q complement/tumor necrosis factor‐associated protein 9 (CTRP9) expression through upregulation of nuclear receptor subfamily 2 group F member 2 (NR2F2). (A) Protocol for screening the transcription factors of CTRP9 by IGF‐1 in isolated cardiomyocytes. (B) IGF‐1 changed the transcriptional profile of cardiomyocytes. (C) Heatmap of the differentially expressed genes between phosphate‐buffered saline (PBS)‐ and IGF‐1‐treated cardiomyocytes. (D) Intersection of transcriptome upregulated genes and predicted transcription factors of CTRP9. (E) Protein levels of NR2F2, FOXP1, and c‐Fos after siRNA silencing. (F) Transcriptional levels of *C1qtnf9* in cardiomyocytes treated with IGF‐1 (*n* = 4). (G) Protein levels of CTRP9 in cardiomyocytes treated with IGF‐1 (*n* = 4). (H) Validation of the binding site of NR2F2 located upstream of *C1qtnf9* (*n* = 3). (I) Cardiac NR2F2 levels in mice with established myocardial infarction (MI) after swimming training for 8 weeks (*n* = 4). (J) Cardiac NR2F2 levels in cardiac‐specific knockdown of CTRP9 mice after exercise (*n* = 4). (K) Cardiac NR2F2 levels in cardiac‐specific overexpression of CTRP9 mice (*n* = 4). (L) Cardiac NR2F2 levels in CTRP9 knockout mice with IGF‐1 administration (*n* = 4). ^*^ *p* < 0.05, ^**^ *p* < 0.01, ^***^ *p* < 0.001.

To investigate the mechanism by which IGF‐1 upregulates cardiac CTRP9 expression, transcriptome analysis was performed, which revealed that IGF‐1 modulated the transcriptional profile of cardiomyocytes (Figures 6A–C and S1). Among the differentially expressed genes between phosphate‐buffered saline (PBS)‐ and IGF‐1‐treated cardiomyocytes, we identified three potential transcription factors of CTRP9 (Figure 6D). Knockdown of these three genes using siRNA showed that the knockdown of *Nr2f2* abolished the effects of IGF‐1 on the upregulation of CTRP9 expression (Figure 6E–G). In addition, dual‐luciferase experiments confirmed that nuclear receptor subfamily 2 group F member 2 (NR2F2) acts as a transcription factor of CTRP9 (Figure 6H). We also detected NR2F2 expression in mice with established MI, showing that low‐dose exercise upregulated cardiac NR2F2 expression in mice with established MI, and found that low‐dose exercise upregulated cardiac NR2F2 expression in these mice (Figure 6I). Moreover, low‐dose exercise upregulated cardiac NR2F2 expression in both control and CTRP9 knockdown mice with established MI and CTRP9 overexpression had little effect on cardiac NR2F2 expression in both sham and MI mice (Figure 6J,K). Additionally, IGF‐1 treatment increased cardiac NR2F2 expression in both WT and CTRP9 KO mice (Figure 6L). Taken together, these results suggest that IGF‐1 upregulates cardiac CTRP9 expression through the upregulation of NR2F2.

## 3 DISCUSSION

**FIGURE 7:**
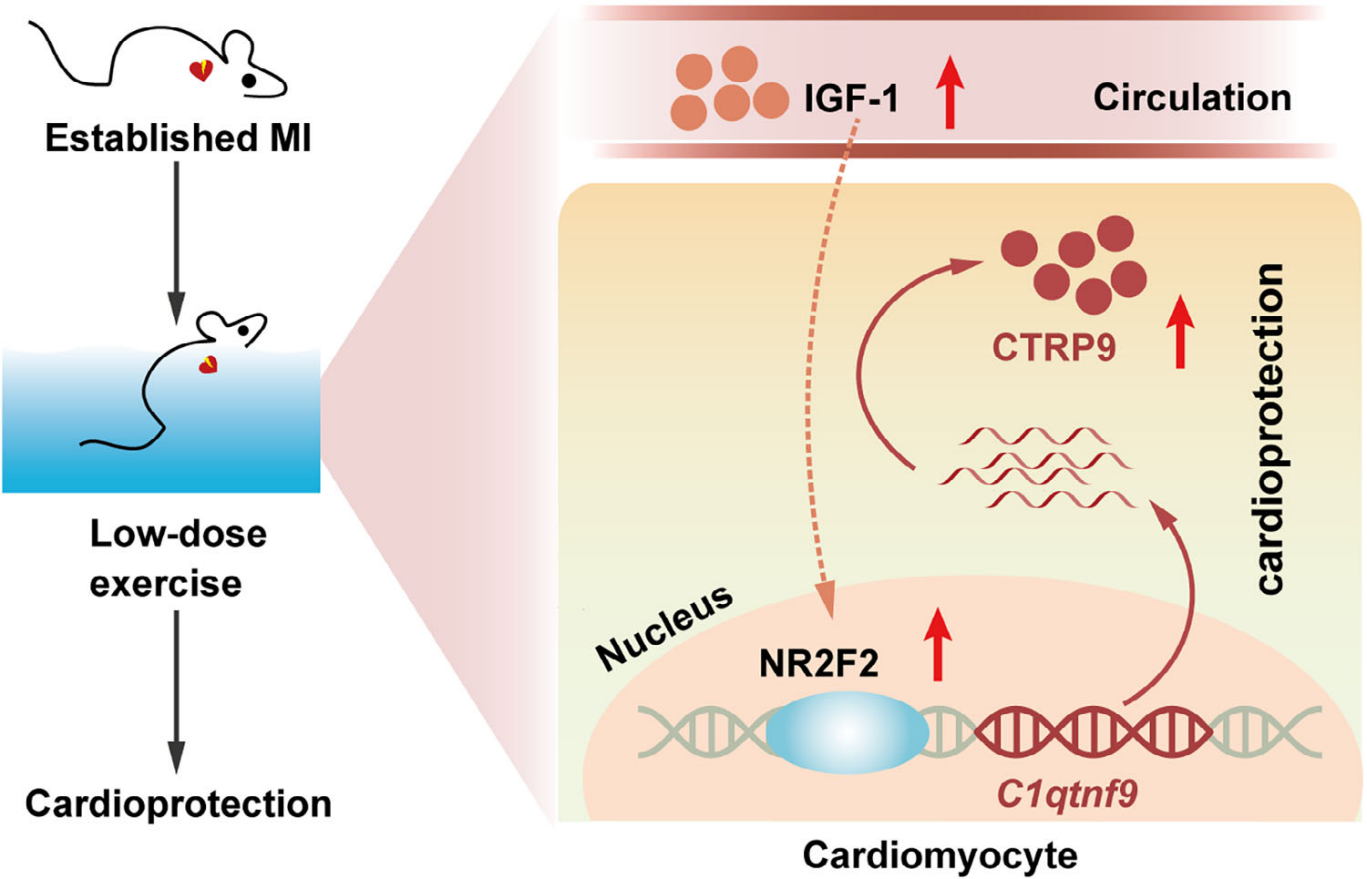
Schematic figure illustrating how low‐dose exercise protects the heart against established myocardial infarction (MI) through consecutive upregulation of cardiac C1q complement/tumor necrosis factor‐associated protein 9 (CTRP9). Low‐dose exercise consecutively increases cardiac CTRP9 expression by upregulating circulating insulin‐like growth factor 1 (IGF‐1), thereby contributing to exercise‐induced cardioprotection against established MI.

Although exercise is recommended as an important component of therapy for CVDs in clinical practice,^5^ there are still major challenges in prescribing an optimized regimen of exercise to individual patients.^7^ For established MI, evidence for an optimized dose of exercise therapy is scarce. In this study, we found that only low‐dose exercise exerted protective effects against established MI, while moderate‐ and high‐dose exercise showed no protective effects, suggesting that a lower dose of exercise should be prescribed for patients with established MI (Figure 7).

Most of the evidence regarding exercise in established heart failure is derived from studies implementing exercise regimes considered safe in stable patients with risk factor control and optimal medical therapy.^15^, ^16^, ^17^, ^18^ Although significant improvements in exercise tolerance and quality of life are reported, the effects of exercise on all‐cause and heart failure‐specific mortality and hospitalization are moderate.^18^, ^19^, ^20^, ^21^, ^22^, ^23^, ^24^ Aerobic exercise at a moderate or lower dose is recommended for patients with established heart failure.^5^ For animal studies, there is mounting evidence showing the benefit of moderate‐intensity exercise on cardiac function in rodents with MI.^25^, ^26^ Most of the studies implement exercise during the progression of heart failure, while evidence for established MI or heart failure is limited.^9^, ^27^ In this study, we showed that only low‐dose exercise significantly improved cardiac structure and function, while moderate‐ and high‐dose exercise did not affect cardiac structure and function in established MI. Additionally, we observed no adverse effects for all doses of exercise in mice with established MI, suggesting that 15−60 min of swimming training every day is safe and well tolerated. However, these results suggest that a safe regime of exercise did not necessarily mean cardiac benefits for established MI.

In this study, divergent impact of low‐dose exercise versus high‐dose exercise on fibrosis was observed. Low‐dose exercise reduced cardiac fibrosis, while high‐dose exercise increased cardiac fibrosis in the border region of the heart in mice with established MI. Cardiac fibrosis induced by MI can be classified into replacement fibrosis and active fibrosis.^28^ In the early stage, replacement fibrosis prevents heart rupture after MI by formatting scar.^29^ The later stage is active fibrosis induced by increased mechanical stress post‐MI, together with hormonal and paracrine mediators.^28^ In this study, mice were subjected to exercise after established MI. Thus, fibrosis observed here was active fibrosis (no intervention was performed in the early MI stage). The decreased fibrosis of the post‐MI heart with low‐level exercise may contribute to the anti‐fibrosis effect of CTRP9. Continuous high‐level exercise failed to increase the protective factor CTRP9, as well as might aggravate cardiac output and increase the mechanical load of the heart, leading to a higher fibrosis. Besides, the cardiac function of the mice in the low‐dose exercise group was higher than that in the high‐dose exercise group, so compensatory myocardial fibrosis may also be reduced.

Clinical evidence has shown that exercise therapy has significant benefits for patients with chronic heart failure, but the underlying mechanisms are not well understood. In this study, we found that CTRP9 is a critical factor that mediates the protective effects of low‐dose exercise on cardiac function in mice with established MI. The CTRP family, including CTRP1, CTRP3, CTRP5, CTRP9, CTRP12, and CTRP13, has been shown in recent studies to influence both the development and progression of coronary artery disease by modulating metabolic pathways, influencing the immuno‐inflammatory response, and regulating cardiovascular functions.^12^ High‐dose exercise (60 min/day) upregulated the transcriptional level of *C1qtnf7*. However, the translation level of CTRP7 was not upregulated by exercise. Obviously, upregulated CTRP7 mRNAs did not translate to protein for unknown reasons. For mice after MI, the amount of exercise did not affect the protein content of CTRP7 in myocardial tissue. At least in our study, CTRP7 is most likely not involved in the mechanism by which exercise affects the prognosis of mice after MI. CTRP9 has been reported to have anti‐inflammatory and anti‐atherosclerosis features, contributing to its cardioprotective effects in cardiac diseases.^30^ Additionally, it reduces vascular smooth muscle cell proliferation and induces vasodilation.^31^, ^32^, ^33^ Other studies have also shown that CTRP9 is involved in the regulation of lipid and glucose metabolism.^34^, ^35^, ^36^ CTRP9 is downregulated in heart during acute MI.^14^ We observed that CTRP9 expression was temporally constant in mice with established MI, and all exercise doses upregulated the expression of cardiac CTRP9. However, only low‐dose exercise consecutively upregulated cardiac CTRP9. Knockdown of cardiac CTRP9 abolished the cardioprotective effects of low‐dose exercise, and overexpression of cardiac CTRP9 exerted cardioprotective effects in mice with established MI. These results suggest that low‐dose exercise protects the heart against established MI through upregulation of cardiac CTRP9 expression.

In our previous study, CTRP9 was administered from 4 h to 6 weeks after MI surgery, and found that mice received CTRP9 supplementation had smaller infarct size, confirming that early intervention of CTRP9 after MI reduces acute cardiac dysfunction and protects damaged myocardium.^14^ In this study, the intervention of CTRP9 levels was performed 4 weeks after MI, suggesting that CTRP9 exerts protective effects against established MI.

Mounting evidence has highlighted the critical role of circulating exerkines in regulating exercise‐induced benefits. In this study, we aimed to investigate whether exercise upregulates cardiac CTRP9 expression through circulating factors. Our findings revealed that exercise upregulated cardiac CTRP9 by increasing circulating IGF‐1. IGF‐1 is a well‐identified exerkine secreted mainly from skeletal muscles, and both strength and endurance exercises have been shown to increase circulating IGF‐1 concentrations.^37^ IGF‐1 promotes cell proliferation and angiogenesis, resists apoptosis, and exerts a cardioprotective effect against MI.^38^, ^39^ We also found that IGF‐1 supplementation increased cardiac CTRP9 expression, and intervention with cardiac CTRP9 expression showed no significant effects on circulating IGF‐1 in mice. Additionally, we identified NR2F2 as a downstream regulator of IGF‐1, which acts as a transcription factor of CTRP9. Previous studies have shown that NR2F2 is involved in tissue homeostasis and maintenance, functioning as a major regulator of cell differentiation, the development of several tissues and organs, and angiogenesis.^40^, ^41^, ^42^, ^43^ It has also been reported that NR2F2 is involved in the regulation of intracellular metabolism in multiple tissues.^40^, ^41^, ^42^, ^43^ The relationship between NR2F2 mutation and cardiac dysfunction has been identified, while the role of NR2F2 in the regulation of cardiac function remains largely unknown.^44^, ^45^ The present study provides evidence that NR2F2 acts as an upstream regulator of CTRP9 in the heart.

It is well known that exercise, particularly appropriate exercise, exerts multifaceted protective effects on the body, including significant cardiovascular protection. In recent years, the concept of exerkines (exercise factors), which are produced by various organs and tissues through autocrine, paracrine, and endocrine actions in response to exercise stimuli, has gained increasing recognition among researchers. These factors exert their effects through different signaling pathways. Interestingly, experimental findings by Yang et al.^46^ have shown that exercise can upregulate the expression of BDNF, thereby exerting cardioprotective effects. Also, another study showed that BDNF generation could limit chronic postischemic heart failure.^47^ This finding is similar to our results regarding the effects of CTRP9, indicating that exercise may upregulate the expression levels of different cytokines in cardiac myocytes through various signaling pathways, ultimately leading to postischemic cardiac protection in postischemic heart. It remains to be explored whether BDNF and CTRP9 exert their exercise‐induced cardioprotective effects with upstream/downstream relationship or independent signaling pathway. Taken together, we found that low‐dose exercise can confer protection against established MI in mice, while moderate‐ and high‐dose of exercise have little protective effect. Mechanistically, low‐dose exercise leads to a sustained increase in cardiac CTRP9 expression through the upregulation of circulating IGF‐1, whereas moderate‐ and high‐dose exercise only upregulates cardiac CTRP9 expression in the short term. These results underscore the critical role of CTRP9 in exercise‐induced cardioprotection and suggest that an optimized dose of exercise should be prescribed for patients with established MI.

## 4 MATERIALS AND METHODS

### 4.1 Animals

Adult male C57BL/6 mice (8 weeks old) weighing 23−28 g were used in the study. The animals were provided by the Experimental Animal Center of the Fourth Military Medical University. CTRP9 KO mice were constructed by eliminating the second exon using CRISPR/Cas9 by the Nanjing Biomedical Research Institute of Nanjing University. All mice were housed in collective cages with free access to water and food. The light/dark cycle (12 h−12 h) and room temperature (22°C–24°C) were controlled. All experimental procedures were conducted according to the National Institute of Health (2010) Guide for the Care and Use of Laboratory Animals and were approved by the Animal Research Ethics Committee of the Fourth Military Medical University.

### 4.2 MI surgery

Mouse anesthesia was induced by placing in an anesthesia induction chamber with oxygen flow (1 L/min) and isoflurane (Jiangsu HFQ Bio‐technology Co., Ltd.) content (3%) was adjusted. After full anesthesia induction, 2% isoflurane was used for anesthetization. MI surgery was performed as previously described.^48^ Briefly, the heart was smoothly and gently exposed through a small hole at the fourth intercostal space, followed by left coronary artery was located, sutured, and ligated at a site about 3 mm from its origin using a 6‐0 silk suture. The heart was immediately placed back followed by manual evacuation of air and closure of muscle and the skin. Sham‐operated mice were subjected to the same procedure with the suture untied.

### 4.3 Adeno‐associated virus infection

Recombinant AAV9 with cTnT promotor carrying CTRP9 or CTRP9 shRNA was constructed by Tsingke Biotechnology Co., Ltd. The sequence of CTRRP9 shRNA (5′−3′) was: CCGG‐GCAGGTGTCTATTACTTTACC‐CTCGAG‐GGTAAAGTAATAGACACCTGC‐TTTTTT. Two weeks after MI or sham operation, 100 μL of adeno‐associated virus (AAV) (1 × 10^12^ drips/mL) was administered via tail intravenous injection. Negative control (AAV‐NC) or shRNA negative control (AAV‐shNC) was injected as a control.

### 4.4 Exercise protocol

The swimming protocol was carried out as previously described.^49^ Briefly, mice in the exercise group were adapted to swimming training with a 10 min session on the first day and then progressively increased to the target duration over 1 week. The mice exercised daily for 5 days a week, for 8 weeks, from 8:00 pm to 10:00 pm in water tanks measuring 60 cm × 90 cm, which were filled with warm water (32°C–35°C) to a depth of 25 cm. After each swimming session, the mice were wiped dry with towels and then placed in a ventilated thermostat (36°C–37°C) until their hair was dry. The mice were then returned to their cages.

### 4.5 Intraperitoneal injection of IGF‐1

Four weeks after MI induction, IGF‐1 (ab9861, Abcam) supplementation was initiated by intraperitoneal injection at a dose of 40 μg/kg/day. IGF‐1 was dissolved in 100 μL of PBS per mouse every day for a total of 8 weeks. Mice in control group were injected with 100 μL PBS.

### 4.6 Assessment of cardiac function

Echocardiography was performed according to a previously described method.^50^ Briefly, mouse anesthesia was induced by 3% isoflurane and maintained by 2% isoflurane. After chest hair was removed, echocardiography was performed with a VisualSonics 2100 echocardiograph (VisualSonics 2100, FUJIFILM VisualSonics, Inc.), and a 400‐MHz transducer was used to record short‐axis views of the LV. All data were measured and calculated using Vevo Lab 3.1.0 software (FUJIFILM VisualSonics, Inc.). Hemodynamics were assessed according to a previously described method.^48^ A 1.4 French micromanometer‐tipped catheter (Millar Instruments) was inserted into the LV via the right carotid artery. After a stabilization period of 2 min, the LVEDP and d*P*/d*t*_max_ were continuously recorded using a PowerLab System (AD Instruments, Inc.).

### 4.7 RNA‐seq sequencing and bioinformatics analysis

Total RNA was extracted using the RNAprep Pure Kit (DP432, TIANGEN Biotech Co., Ltd.) following the manufacturer's instructions. RNA‐seq sequencing and bioinformatics analysis were performed by Standard Sci‐Tech Innovation (Qingdao) Pharmaceutical Technology Co., Ltd. according to a previously described method.^51^ Briefly, the integrity of all RNA samples was assessed using the Qsep1 instrument (Bioptic). To construct RNA libraries with the VAHTS mRNA‐seq V3 Library Prep Kit (NR604, Vazyme) for Illumina, 1 μg of total RNA was used.^52^ After routine bioinformatics analysis of the raw data, upregulated genes were identified with edgeR with a filter threshold of fold change (FC) >1.2. The transcription factor of *C1qtnf9* was predicted via Animal TFDB.

### 4.8 Proteomics and bioinformatics analysis

Proteomics and bioinformatics analysis were performed by Standard Sci‐Tech Innovation (Qingdao) Pharmaceutical Technology Co., Ltd. according to a previously described method.^53^ Briefly, serum samples were centrifuged, and the supernatant concentration of the protein was determined using the bicinchoninic acid (BCA) method with the BCA Protein Assay Kit (P0010, Beyotime Biotechnology). After protein denaturation, reduction, alkylation, tryptic digestion, and peptide cleanup, the digested peptides were desalted, and the peptide sample was loaded and separated with a 120 min gradient using Orbitrap QE HF coupled to an EASY‐nanoLC 1200 system (Thermo Fisher Scientific) under data‐independent acquisition (DIA) mode. The raw data of DIA were processed and analyzed using the Spectronaut software (version 11.0, Biognosys) with default settings. Data extraction was determined by Spectronaut software (version 11.0, Biognosys) based on extensive mass calibration. Differentially expressed proteins were selected using Student's *t*‐test if their FC ≥ 1.5.

### 4.9 Tissue collection

Animals anesthetized by isoflurane were sacrificed, and their hearts were removed immediately after blood collection. Serum samples were collected after blood was allowed to settle for 40 min at 4°C. The mouse hearts were either stored at −80°C for western blotting and quantitative real‐time polymerase chain reaction (qPCR) or fixed in 4% paraformaldehyde for histological analysis.

### 4.10 qPCR

Total RNA was extracted using the Total RNA extraction kit (TIANGEN) following the manufacturer's instructions. The CFX96 real‐time PCR system‐C1000 Thermal Cycler (Bio‐Rad Laboratories) was used for qPCR using StarLighter SYBR Green qPCR Mix (FS‐Q1002, Forever Star) after reverse‐transcription of RNA into complementary DNA using StarLighter Script RT all‐in‐one Mix (FS‐P1002, Forever Star). Transcription levels of target genes were quantified using the 2^−ΔΔCt^ method and normalized to *Actb*.^54^ The primer sequences were synthesized by Tsingke Biotechnology Co., Ltd. and are listed in Table S1.

### 4.11 Isolation and culture of neonatal rat cardiomyocytes

Neonatal rat cardiomyocytes (NRCMs) were obtained from the ventricles of newborn Sprague–Dawley rats. The isolation and resuspension protocol for primary NRCMs was as previously described.^55^ The resuspended NRVMs were then inoculated in the cell culture flasks or dishes after differential adhesion for subsequent experiments. NRCMs were cultured in F12 Dulbecco's modified Eagle medium (11320033, Thermo Fisher Scientific) supplemented with 10% fetal bovine serum (10091148, Thermo Fisher Scientific), 1% penicillin–streptomycin solution (15070063, Thermo Fisher Scientific) to a final concentration of 100 U/mL penicillin, and 100 μg/mL streptomycin at 37°C with 5% CO_2_. Depending on the experimental grouping, IGF‐1 (50437, Sino Biological Inc.) or HABP2 (HY‐P70832, MedChemExpress LLC) was added 24 h before the end of the culture.

### 4.12 Western blot

Total proteins were isolated from NRCMs or heart tissues using a lysis buffer containing protease inhibitor (11836170001, Roche). The protein concentration was determined using a BCA protein assay kit (P0012, Beyotime). Proteins were separated via gel electrophoresis, using a prestained protein ladder (SW175‐04, Sevenbio) as an indicator, transferred to a polyvinylidene fluoride membrane, and blocked with 5% fat‐free milk. The membranes were then incubated with primary antibodies including CTRP2 (LS‑C145229, Lifepan), CTRP7 (ABN‐PAB13422, Abnova), CTRP9 (customized by Genscript), α‐SMA (ab5694, Abcam), TGF‐β (ab92486, Abcam), β‐MHC (sc‐53089, Santa Cruz), c‐Fos (2250T, CST), FOXP1 (ab134055, Abcam), NR2F2 (ab211777, Abcam), and β‐actin (AT0001) and GAPDH (AT0002) purchased from cmcTAG. The secondary antibodies used were goat anti‐rabbit (ZB‐2301) and goat anti‐mouse (ZB‐2305) purchased from Zhongshan Company. The blots were visualized using ChemiDoc XRS (Bio‐Rad Laboratories). The gray value of protein bands was analyzed using Image Lab 5.1 software (Bio‐Rad Laboratories). β‐Actin or GAPDH was used as the internal control to normalize the protein expression.

### 4.13 Histological analysis

Heart tissues were fixed in 4% paraformaldehyde for 24 h, followed by embedding and sectioning. The sections were stained with hematoxylin and eosin (Sigma–Aldrich) according to the manufacturer's instructions. Masson staining and wheat germ agglutinin (WGA) immunofluorescence were performed as previously described.^50^ The infarction size was determined by Masson staining and digitalized, and the analysis was conducted using ImageJ software (NIH). The fibrosis area and WGA staining were processed with ImageJ software.

### 4.14 Statistics

Data are presented as the mean ± SEM. Data were compared using two‐tailed Student's *t*‐test for two groups, one‐way analysis of variance (ANOVA) with Tukey's multiple comparisons test for three or more groups, or two‐way ANOVA with Tukey's multiple comparisons test for multiple parameters after normality assumptions were confirmed by the Shapiro–Wilk test. Non‐parametric Mann–Whitney or Kruskal–Wallis tests were used when normality assumptions were rejected. The log‐rank (Mantel–Cox) method was used to compare survival curves. Statistical analyses were performed in GraphPad Prism v8.0.

## AUTHOR CONTRIBUTIONS

W.Y., D.Y., and Y.S. designed the study. J.L., W.D., and J.Y. were responsible for reviewing the data. E.G. performed all animal surgeries. Y.T., P.F., L.F., L.S., and Y.S. implemented the experiments and collected data. B.Z. and Y.T. analyzed the data. B.Z., Y.T., and P.F. drafted the manuscript. All authors revised and approved the final manuscript.

## CONFLICT OF INTEREST STATEMENT

The authors declare they have no conflicts of interest.

## ETHICS STATEMENT

All experimental procedures were performed in adherence with the National Institutes of Health Guidelines for the Use of Laboratory Animals and were approved by the Fourth Military Medical University Committee of Animal Care (No. IACUC‐20190350).

## Supporting information

Supporting Information

## Data Availability

All data used and/or analyzed during the current study are available from the corresponding author upon reasonable request.
