# Supplementary material for: Low‐dose exercise protects the heart against established myocardial infarction via IGF‐1‐upregulated CTRP9 in male mice
Source: MedComm (2020). 2023 Nov 24;4(6):e411. doi: 10.1002/mco2.411 (PMC10674078; doi:10.1002/mco2.411)
Supplement: Supplementary file 1 — Supporting Information [file MCO2-4-e411-s001.docx]

**Low-dose exercise protects the heart against established myocardial infarction via IGF1-upregulated CTRP9 in male mice**

**Runnting title:** Low-dose exercise protects MI heart via CTRP9

Yanzhen Tan^1,#^, Pan Feng^1,#^, Lele Feng^1^, Lei Shi^1^, Yujie Song^1^, Jian Yang^1^, Weixun Duan^1^, Erhe Gao^3^, Jincheng Liu^1^, Dinghua Yi^1^, Bing Zhang^1,^*, Yang Sun^2,^*, Wei Yi^1,^*

1 Department of Cardiovascular Surgery, Xijing Hospital, Fourth Military Medical University, Xi’an 710032, China.

2 Department of General Medicine, Xijing Hospital, Fourth Military Medical University, Xi’an 710032, China.

3 Center for Translational Medicine, Lewis Katz School of Medicine at Temple University, 19140 Philadelphia, PA, USA.

^#^ Yanzhen Tan and Pan Feng contributed equally to this study.

*Corresponding author:

Wei Yi, Department of Cardiovascular Surgery, Xijing Hospital, Fourth Military Medical University, No.127, Changlexi Road, Xi'an, Shaanxi, 710032, China. Email: yiwei@fmmu.edu.cn

Yang Sun, Department of General Medicine, Xijing Hospital, Fourth Military Medical University, No.127, Changlexi Road, Xi'an, Shaanxi, 710032, China. Email: drsunyang@fmmu.edu.cn

Bing Zhang, Department of Cardiovascular Surgery, Xijing Hospital, Fourth Military Medical University, No.127, Changlexi Road, Xi'an, Shaanxi, 710032, China. Email: bingzhang66@fmmu.edu.cn

Present address of Pan Feng: Department of Cardiology, The 305 Hospital of People’s Liberation, Beijing, 100017, China

**
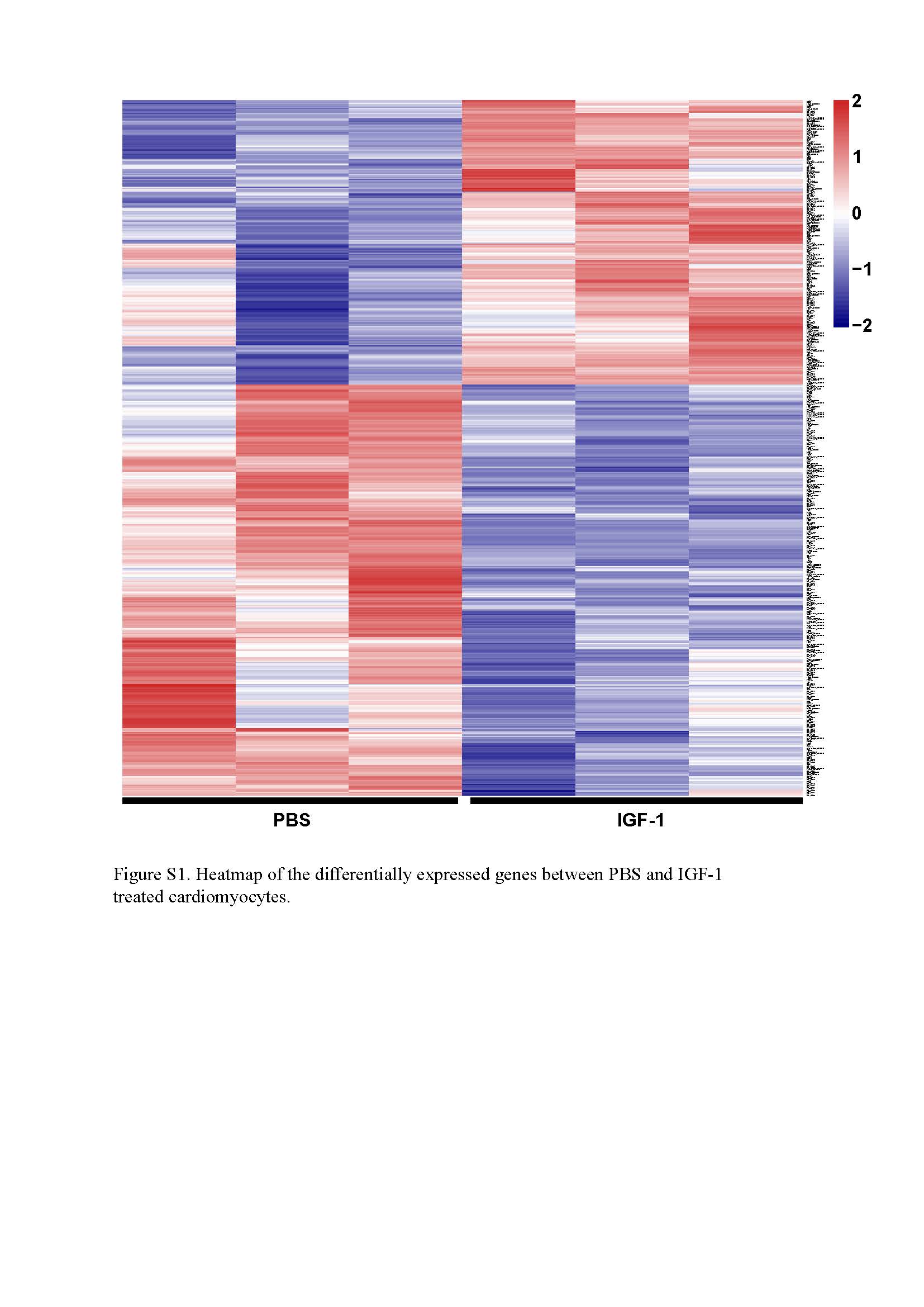
**

**Figure S1. Heatmap of the differentially expressed genes between PBS and IGF-1**

**treated cardiomyocytes.**

**TABLE S1. A list of the primers used in the RT- PCR**

| **Gene** | **Species** | **Forward primer** | **Reverse primer** |
| --- | --- | --- | --- |
| *Actb* | Mouse | CACTGTCGAGTCGCGTCC | CGCAGCGATATCGTCATCCA |
| *C1qtnf1* | Mouse | GGAGGATTTCATTGCACAGCC | CAGCATCCCAGCATGAATCC |
| *C1qtnf2* | Mouse | CCCTTATTGGACCGACAGCC | CAGACGAACTTGCCACTGGA |
| *C1qtnf4* | Mouse | TGTCCTTCCCACTTGCTCAC | CTACACACTGGTCGCCCG |
| *C1qtnf5* | Mouse | CTTGGCCTCTGAACAACTGGT | TAGTCTGTAGGCCCTTCCCG |
| *C1qtnf6* | Mouse | GTTTCCCCTTATGTCCTGCCTG | TTCACAAAGACCCTGGCTTCCC |
| *C1qtnf7* | Mouse | GGGAAGGATCGCAGTTGCTTTA | TCTGCAACCGTCTTTCTCAGT |
| *C1qtnf9* | Mouse | TTTGGTGGCTTCTGCTGGTT | AGGTAGGCCATTGTGACCTG |
| *C1qtnf12* | Mouse | GAGAGCAACAGCAGGGTCTT | CCCAGAACTGTTGTCCACGA |
| *Actb* | Rat | CTGTGTGGATTGGTGGCTCT | CAGCTCAGTAACAGTCCGCC |
| *C1qtnf9* | Rat | ACTGAAGCTTGGGGACGAAG | ACGTGCAGAGTCCTTTGTGT |
| *Nr2f2* | Rat | CAATCAACTAGCCCTGAGCCA | TGCCATATCTATGGGGGCCG |
| *Foxp1* | Rat | CAAGAATCTGGGTCTGAGGCA | CAAGAATCTGGGTCTGAGGCA |
| *Fos* | Rat | ACGACCATGATGTTCTCGGG | CGGACAGATCTGCGCAAAAG |
